# Characterization of Fitness Cost Caused by Tigecycline-Resistance Gene *tet*(X6) in Different Host Bacteria

**DOI:** 10.3390/antibiotics10101172

**Published:** 2021-09-27

**Authors:** Lijie Jiang, Wenhui Cai, Feifei Tang, Zhiqiang Wang, Yuan Liu

**Affiliations:** 1 College of Veterinary Medicine, Yangzhou University, Yangzhou 225009, China; lijieo@163.com (L.J.); cwh15248179347@163.com (W.C.); tff96324@163.com (F.T.); 2 Jiangsu Co-Innovation Center for Prevention and Control of Important Animal Infectious Diseases and Zoonoses, Joint International Research Laboratory of Agriculture and Agri-Product Safety, The Ministry of Education of China, Yangzhou University, Yangzhou 225009, China; 3 Institute of Comparative Medicine, Yangzhou University, Yangzhou 225009, China

**Keywords:** tigecycline, *tet*(X6), host strains, fitness cost, virulence

## Abstract

The emergence and prevalence of the *tet*(X) gene and its variants in the environment and in clinical settings constitute a growing concern for public health worldwide. Accordingly, the tigecycline resistance gene variant *tet*(X6) is widely detected in *Proteus* spp. and *Acinetobacter* spp. rather than Enterobacteriaceae, while the underpinning behind this phenomenon is still unclear. To investigate the mechanisms underlying this distinct phenomenon, we assessed the fitness of the engineered plasmid pBAD-*tet*(X6) in different host bacteria by monitoring their growth curves, relative fitness and the ability of biofilm formation, as well as virulence in a *Galleria mellonella* model. MIC and qRT-PCR analysis indicated the successful expression of the *tet*(X6) gene in these strains in the presence of l-arabinose. Furthermore, we found that pBAD-*tet*(X6) displayed the lowest fitness cost in *P. mirabilis* compared with that in *E. coli* or *S.* Enteritidis, suggesting the fitness difference of *tet*(X6)-bearing plasmids in different host bacteria. Consistently, the carriage of pBAD-*tet*(X6) remarkably reduced the biofilm production and virulence of *E. coli* or *S.* Enteritidis. These findings not only indicate that the fitness cost difference elicited by the *tet*(X6) gene may be responsible for its selectivity in host bacteria but also sheds new insight into the dissemination of antibiotic resistance genes (ARGs) in clinical and environmental isolates.

## 1. Introduction

Antimicrobial resistance is a growing global threat to public health and human safety [1]. Tigecycline is a broad-spectrum glycosyl cyclopeptide antibiotic, belonging to the third generation of tetracycline antibiotics [2,3]. It is recognized as the last line of defense for the treatment of multidrug-resistant (MDR) bacterial infections, particularly for carbapenem-resistant Enterobacteriaceae (CRE). However, with the emergence of plasmid-mediated high-level tigecycline resistance genes *tet*(X3/X4), the risk of treatment failure for MDR bacteria is increasing dramatically [4,5]. Moreover, the *tet*(X) gene can also be transferred horizontally by conjugative plasmids between intra- and inter-species. Enterobacteriaceae were revealed as one of the important hosts for the *tet*(X) gene, particularly for the *tet*(X4) gene [6,7]. Recently, a novel *tet*(X) variant named *tet*(X6) was frequently detected from *Proteus* spp. [8] and *Acinetobacter* spp. [9], rather than from *Escherichia coli*, *Salmonella* spp. and other commonly occurring bacteria. However, the mechanisms by which these *tet*(X6) plasmids have better adaptability in *Proteus* spp. remain unclear.

Previous studies suggest that antibiotic resistance is commonly accompanied by a biological fitness cost for resistant bacteria, expressed as, e.g., reduced growth rates, lower virulence or decreased transmission [10,11]. For example, the increased expression of the mobile colistin resistance gene (*mcr-1*) has led to a decreased growth rate, cell viability and lower production of IL-6 and TNF, as well as alleviated virulence in the *Galleria mellonella* infection model [12]. Nevertheless, processes such as compensatory evolution, cost-free resistance and co-selection between the resistance mechanism and other selected markers can affect the dynamics of bacterial resistance spread within the population, both in the presence and absence of antibiotic pressure, and sometimes allow the resistance mechanism to establish itself in a bacterial population [13,14]. Recently, Yang et al. identified a putative ProQ/FinO family protein named PcnR, which regulated the expression of the *mcr-1* gene and bacterial fitness by inhibiting the copy number of IncI2 plasmids [15]. A comprehensive investigation of fitness costs in antibiotic-resistant bacteria would be helpful to forecast the development of antibiotic resistance.

Given that the *tet*(X6) gene is rarely found in Enterobacteriaceae, we hypothesized that the existence of *tet*(X6) in *Proteus* spp. may be accompanied by a lower fitness cost compared with Enterobacteriaceae such as *E. coli* or *S.* Enteritidis. To test this hypothesis, we evaluated the fitness of the engineered plasmid pBAD-*tet*(X6) in different host bacteria by monitoring their growth curve, relative fitness, the ability of biofilm formation and virulence in a *Galleria mellonella* model. Deciphering the underlying mechanisms of action would be conducive to understanding the dissemination of *tet*(X6) genes in clinical and environmental samples and developing effective countermeasures.

## 2. Materials and Methods

### 2.1. Bacterial Strains and Plasmids

Three bacterial strains, including *Escherichia coli* TOP10, *Salmonella* Enteritidis ATCC 13076 and *Proteus mirabilis* HS1-T, were used in this study. Unless otherwise stated, bacteria were grown in LB broth or LB agar at 37 °C. To test the effect of *tet*(X6) expression on bacterial growth, the gene encoding the Tet(X6) resistance protein was cloned into a pBAD-HisA vector using the optimized primers (Table 1). All resultant plasmids were transformed into *E. coli* TOP10, *S.* Enteritidis ATCC 13076 and *Proteus mirabilis* HS1-T. The positive transformants were screened by agar plate containing tigecycline (2 μg/mL) and verified by PCR analysis.

### 2.2. Quantitative Real-Time PCR

Relative expression of *tet*(X6) gene in different host bacteria was determined by a two-step qRT-PCR analysis using primers q*tet*(X6)-F, q*tet*(X6)-R and 16S rRNA as the reference gene (Table 1). Briefly, total RNA was extracted from bacteria using the RNA-easy Isolation Reagent (Vazyme, Nanjing, China), followed by complementary DNA synthesis with DNA integrated genomic DNA (gDNA) removal using reverse transcription kit. Relative expression results were calculated using the 2^−ΔΔCt^ method.

### 2.3. Effects of tet(X6) Overexpression on Bacterial Growth

An overnight culture of above strains was diluted 10-fold appropriately, and these strains were exposed to different l-arabinose concentrations from 0 to 10 mM and incubated at 37 °C. Bacteria counts were determined at 0, 3, 5, 7 and 9 h by subjecting to 10-fold serial dilution and then plating onto MH agar for visible counts calculation. Meanwhile, the absorbance of culture at 600 nm was determined using an Infinite M200 Microplate reader (Tecan, Männedorf, Switzerland) [16].

### 2.4. In Vitro Competition and Biofilm Formation

*In vitro* competition experiment [12] was used to measure the relative fitness of the *E. coli* TOP10 (*tet*(X6)/pBAD) and *S.* Enteritidis ATCC13076 (*tet*(X6)/pBAD) and *P. mirabilis* HS1-T (*tet*(X6)/pBAD). These strains were competed against their parental strains *E. coli* TOP10, *S.* Enteritidis ATCC13076 and *P. mirabilis* HS1-T, respectively. In biofilm formation test, bacterial cells were stained with crystal violet and the absorbance at 590 nm was measured based on previous study [17]. Blank LB broth was used as negative control.

### 2.5. Galleria mellonella Infection Model

In vivo virulence was evaluated using a *G. mellonella* infection model [18]. The wax moth *G. mellonella* in larval stage was stored in dark and used within 3 days. Prior to inoculation into larvae, bacterial pellets were washed with sterile saline and then diluted into an appropriate cell density. Then, the larvae (*n* = 10 per group) were infected with either bacterial suspension (10 μL, 10^7^ CFUs per larvae) pre-incubated with different concentrations of l-arabinose or vehicle at the left posterior gastropoda using a 50 μL Hamilton syringe.

### 2.6. Statistical Analysis

Statistical analysis was performed using GraphPad Prism, version 8.3.0. All data were presented as the mean ± SD from three biological replicates. *p* values were determined using an unpaired, two-tailed Student’s *t*-test.

## 3. Results and Discussion

In this study, three kinds of strains were used as host cells, including *E. coli* TOP10, *S.* Enteritidis ATCC13076 and *P. mirabilis* HS1-T. The *E. coli* TOP10 and *S.* Enteritidis ATCC13076 are standard strains and have been widely used in previous studies [19,20]. The wild-type *P. mirabilis* strains were isolated and identified by our laboratory [21], which were naturally resistant to tigecycline. In order to ensure that the genetic background of the strains was clear, *P. mirabilis* were sequenced and those without the *tet*(X6) gene were chosen. Strain HS1-T was sensitive to ampicillin, which meets the requirement for subsequent transformants screening. Considering these points, *P. mirabilis* HS1-T was selected as the host bacteria.

To validate the expression of *tet*(X6) gene in these strains, MIC values were determined by a broth micro-dilution method in accordance with CLSI guidelines [22]. All media were freshly prepared in order to minimize the oxidative degradation of tigecycline. The MICs for tigecycline ranged from 0.125 to 4 μg/mL in the uninduced group and 1 to 8 μg/mL in the presence of 10 mM l-arabinose (Table 2). Specifically, the MIC values of *E. coli* TOP10 (*tet*(X6)/pBAD) and *S.* Enteritidis ATCC13076 (*tet*(X6)/pBAD) were significantly increased 8-fold after being incubated with l-arabinose (10 mM), implying the successful expression of *tet*(X6) gene in these strains. Interestingly, only two-fold MIC changes in *P. mirabilis* HS1-T (*tet*(X6)/pBAD) were observed. We reasoned that the intrinsic resistance of *P. mirabilis* to tigecycline affects the appearance of the *tet*(X6)-mediated resistance phenotype.

**Figure 1:**
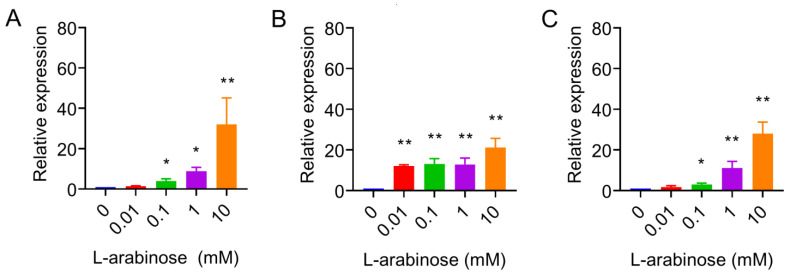
Relative expression level of *tet*(X6) gene in *E. coli* TOP10 (**A**), *S.* Enteritidis ATCC13076 (**B**) and *P. mirabilis* HS1-T (C) in the presence of l-arabinose ranging from 0 to 10 mM. Data are representative of three biological replicates and expressed as mean ± SD. The differences in *tet*(X6) expression were determined using two-tailed Student’s *t*-test (* *p* < 0.05 and ** *p* < 0.01).

Next, the relative expression of the *tet*(X6) gene under the exposure of increasing concentrations of l-arabinose from 0 to 10 mM was monitored using qRT-PCR analysis. The mRNA expression of the *tet*(X6) gene was significantly increased in these three tested strains in a dose-dependent manner (Figure 1), indicating that the expression vector in three strains, particularly for *P. mirabilis* HS1-T, was successfully constructed. Interestingly, when the concentration of l-arabinose was lower than 1 mM, the *tet*(X6) expression level in *E. coli* TOP10 and *P. mirabilis* HS1-T was low, similar to the uninduced group. In contrast, 1 mM l-arabinose resulted in a significantly increased expression of the *tet*(X6) gene in *S.* Enteritidis ATCC13076, indicating that *S.* Enteritidis is more sensitive to the stimulation of l-arabinose at low levels.

**Figure 2:**
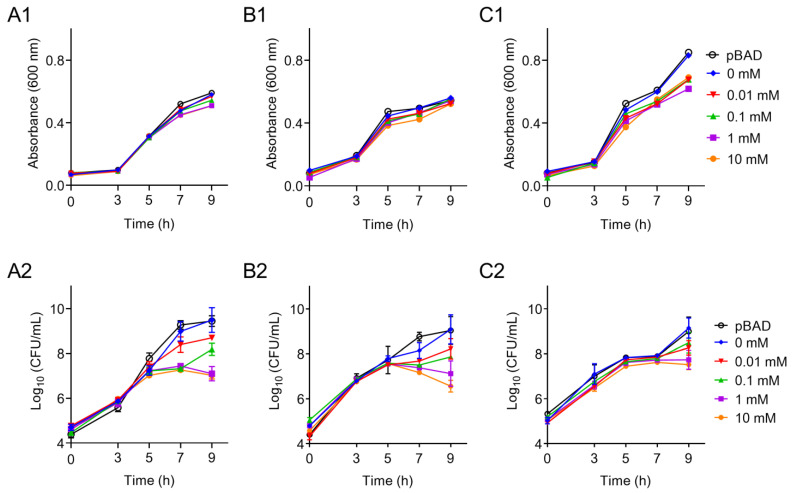
Effect of *tet*(X6) overexpression on bacterial growth in different host bacteria. (**A1**,**A2**): effect of *tet*(X6) expression on bacterial growth induced with different concentrations of l-arabinose in *E. coli* TOP10. (**B1**,**B2**): effect of *tet*(X6) expression on bacterial growth induced with different concentrations of l-arabinose in *S.* Enteritidis ATCC13076. (**C1**,**C2**): effect of *tet*(X6) expression on bacterial growth induced with different concentrations of l*-arabinose* in *P. mirabilis* HS1-T. (**A1**–**C1**), bacterial growth was represented by absorbance at 600 nm; (**A2**–**C2**), bacterial growth was represented by bacterial CFUs. Host bacteria carrying pBAD plasmid without *tet*(X6) gene were used as a control. Data are representative of three biological replicates and expressed as mean ± SD.

According to the bacterial growth curves, we found that the OD_600_ of all three host strains increased and reached the peak value at 9 h (Figure 2A1–C1). However, no significant difference between the control and l-arabinose treated groups was observed. Considering that the absorbance of culture could not distinguish the live and dead bacteria, we next determined the bacterial CFUs using a plate counting method. Consequently, we found that the addition of l-arabinose remarkably decreased the colony quantity of *E. coli* TOP10 (*tet*(X6)/pBAD) and *S.* Enteritidis ATCC13076 (*tet*(X6)/pBAD) in a dose-dependent manner after 7 h of culture (Figure 2A2–B2). However, the overexpression of the *tet*(X6) gene even under the stimulation of high levels of l-arabinose showed little effect on HS1-T (*tet*(X6)/pBAD) (Figure 2C2). These data indicated that the increased expression of the *tet*(X6) gene will pose an extra burden on the bacteria growth of Enterobacteriaceae rather than the *Proteus* species. These results were consistent with previous findings that the overexpression of antibiotic resistance genes (ARGs) would inhibit the growth of certain bacteria and even lead to the death of bacteria [23,24]. We supposed that there are some regulation mechanisms or compensatory evolution that exist in *P. mirabilis*. These unknown mechanisms lower the fitness cost induced by the *tet*(X6) gene and may promote the epidemic of antibiotic resistance [25].

**Figure 3:**
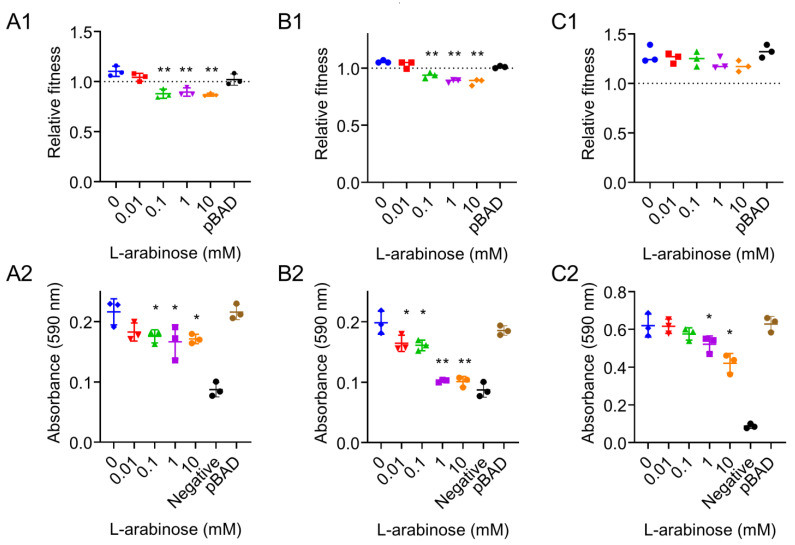
Bacterial fitness cost (**A1**–**C1**) and the ability of biofilm formation (**A2**−**C2**) in different host bacteria after exposure to different concentrations of l-arabinose. (**A1**,**A2**): effect of *tet*(X6) expression on bacterial fitness cost and biofilm formation in *E. coli* TOP10. (**B1**,**B2**): effect of *tet*(X6) expression on bacterial fitness cost and biofilm formation in *S.* Enteritidis ATCC13076. (**C1**,**C2**): effect of *tet*(X6) expression on bacterial fitness cost and biofilm formation in *P. mirabilis* HS1-T. Blank LB broth was used as negative control. Host bacteria carrying pBAD plasmid without *tet*(X6) gene were also determined. Data are representative of three biological replicates and expressed as mean ± SD. The *p* values (* *p* < 0.05 and ** *p* < 0.01) were calculated by two-tailed Student’s *t*-test.

In vitro competition experiments showed that the relative fitness of *P. mirabilis* HS1-T (*tet*(X6)/pBAD) is more than one, regardless of the addition of l-arabinose, suggesting that the *tet*(X6) expression imposed no fitness cost for *P. mirabilis* (Figure 3C1). By contrast, a decreased competitive ability was found in *E. coli* TOP10 (*tet*(X6)/pBAD) and *S.* Enteritidis ATCC13076 (*tet*(X6)/pBAD) when the l-arabinose concentration was above 0.1 mM (Figure 3A1,B1). Previous studies have reported that bacterial biofilm production is related to its survival status, thus, the ability of biofilm formation can also reflect bacteria fitness [26,27]. A crystal violet staining method was applied to evaluate the ability of biofilm formation in different host bacteria carrying the *tet*(X6) gene. Consistent with the relative fitness results, *P. mirabilis* HS1-T (*tet*(X6)/pBAD) displayed the highest biofilm formation ability among them by more than two-fold than the other two strains (Figure 3A2–C2). These results support our idea that the expression of the *tet*(X6) gene in Enterobacteriaceae such as *E. coli* and *S.* Enteritidis rather than *P. mirabilis* would affect bacterial biofilm formation and pathogenicity. Combined with the growth characteristics of strains, we concluded that the overexpression of the *tet*(X6) gene resulted in the remarkable fitness cost in Enterobacteriaceae. This also means that drug-resistant Enterobacteriaceae will lose their competitive advantage over sensitive strains in the absence of antibiotics [28]. By contrast, a balance was achieved in *P. mirabilis* between the fitness cost and *tet*(X6) gene expression. Further studies are warranted to reveal the mechanisms underlying how *P. mirabilis* maintains the *tet*(X6) resistance gene without an obvious fitness cost.

**Figure 4:**
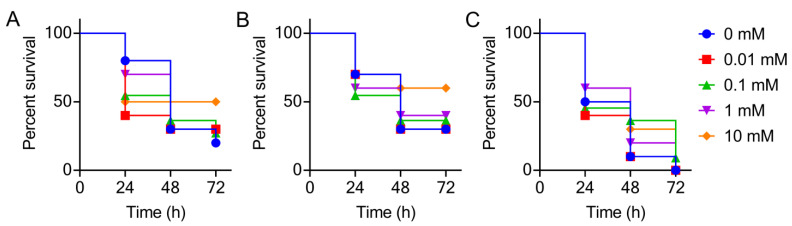
Kaplan–Meier plots showing the percentage of survival of *G. mellonella* over 72 h, infected with different host bacteria induced by different concentrations of l-arabinose. Survival rate of *G. mellonella* larvae (*n* = 10 per group) infected with *E. coli* TOP10 (*tet*(X6)/pBAD) (**A**) and *S.* Enteritidis ATCC13076 (*tet*(X6)/pBAD) (**B**) and *P. mirabilis* HS1-T (*tet*(X6)/pBAD) (**C**) (10^7^ CFUs per larvae) over 72 h.

It is suggested that the fitness cost caused by antibiotic resistance may lead to the reduction of virulence in bacteria, such as in *P. aeruginosa* [29]. In our study, a significant increase in the survival of *G. mellonella* larvae was also observed in *E. coli* TOP10- and *S.* Enteritidis ATCC13076 transformants-infected groups as increased concentrations of l-arabinose (Figure 4). However, the *P. mirabilis* HS1-T transformant retained its pathogenicity on larvae despite the expression of the *tet*(X6) gene. This could also be attributed to a difference in the growth rates of these strains in vivo; the relatively fast growth of *P. mirabilis* HS1-T transformant resulted in a more rapid increase in the bacterial burden in the animal infection model compared with *E. coli* TOP10 and *S.* Enteritidis ATCC13076 transformants.

## 4. Conclusions

In summary, our findings reveal that the carrying and expression of *tet*(X6) gene would result in significant fitness costs in *E. coli* and *S.* Enteritidis rather than in *P. mirabilis*, which may explain the clinical phenomenon that most *tet*(X6) genes were isolated from *Proteus* spp. but not from Enterobacteriaceae. The possible reasons may be attributed to some specific regulator mechanisms in *Proteus* spp., which balances the fitness cost caused by the expression of *tet*(X6) gene and deserves further studies.

## Figures and Tables

**Table 1:** PCR primers used in this study.

Genes	Sequences (5′ to 3′)	Amplicon Size (bp)
*tet*(X6)	F: CGAGCTCATGACTTTACTAAAACATAAAAAAATTAC R: CCCAAGCTTTTATAGATTCATTAGTTTTTGGAAAGAA	1149
qPCR-*tet*(X6)	F: TGTCGTTGATTTTCTCCTG R: TTGATTCTGCCTGTGCTT	332
16S rRNA	F: TTCGGGAACCGTGAGA R: CTGGCAACAAAGGATAAGG	103

The underlined sequence represents restriction enzyme cutting site.

**Table 2:** MIC values of tigecycline for three engineered strains in the absence and presence of l-arabinose.

Strains	MIC (μg/mL)	MIC (μg/mL) with l-Arabinose (10 mM)
*E. coli* TOP10 (*tet*(X6)/pBAD)	0.125	1
*S.* Enteritidis ATCC13076 (*tet*(X6)/pBAD)	1	8
*P. mirabilis* HS1-T (*tet*(X6)/pBAD)	4	8

## Data Availability

All data in this study have been included in this manuscript.
